# Expression of anti-amyloid CARs in microglia promotes efficient and selective phagocytosis of Aβ1‒42

**DOI:** 10.1038/s41434-025-00534-9

**Published:** 2025-04-10

**Authors:** Christina N. Heiss, Rebecca Riise, Eric Hanse, Stefanie Fruhwürth, Henrik Zetterberg, Andreas Björefeldt

**Affiliations:** 1 Department of Psychiatry and Neurochemistry, Institute of Neuroscience and Physiology, University of Gothenburg, Gothenburg, Sweden; 2 Sahlgrenska Center for Cancer Research, Institute of Clinical Sciences, University of Gothenburg, Gothenburg, Sweden; 3 Department of Physiology, Institute of Neuroscience and Physiology, University of Gothenburg, Gothenburg, Sweden; 4 Clinical Neurochemistry Laboratory, Sahlgrenska University Hospital, Mölndal, Sweden; 5 Department of Neurodegenerative Disease, UCL Institute of Neurology, London, UK; 6 UK Dementia Research Institute at UCL, London, UK; 7 Hong Kong Center for Neurodegenerative Diseases, Hong Kong, China; 8 Wisconsin Alzheimer’s Disease Research Center, University of Wisconsin School of Medicine and Public Health, University of Wisconsin-Madison, Madison, WI USA

**Keywords:** Neuroscience, Neurological disorders

## Abstract

Genetic engineering of microglial cells is a promising therapeutic avenue emerging with advancements in gene delivery techniques. Using a recently developed AAV capsid for efficient in vitro transduction we report the engineering of microglia with CARs (CAR-Mic) targeting phagocytosis of amyloid beta 1‒42 (Aβ42). Functional screening of seven CAR constructs in human iPSC-derived microglia revealed up to 6-fold increases in internalized Aβ relative to viral control. CAR-driven phagocytic enhancement was selective for Aβ, dependent on intracellular domain signaling, and was confirmed in primary mouse microglia. These findings highlight the potential of using this approach to target dysfunctional microglia in Alzheimer’s disease and other CNS disorders.

## Introduction

Microglia are central nervous system (CNS)-specialized immune cells with critical roles in neural development, tissue surveillance and homeostasis. Substantial evidence point to their dysfunction being a key contributor to pathology in multiple neurodegenerative diseases (NDDs) [1], advocating for development of advanced microglia-based therapies [2, 3]. In late onset Alzheimer’s disease (AD), a prototypical NDD accounting for ˃95% of AD cases, microglia gradually lose early protective functions such as phagocytosis of amyloid beta (Aβ) and become increasingly pro-inflammatory. Recent human genetic studies show that AD risk genes are highly enriched in microglia and converge on pathways involved in Aβ clearance and degradation [4, 5]. This, together with clinical trial results indicating microglial involvement in Aβ removal in recently approved anti-amyloid antibody treatments [6, 7], suggests that strategies to enhance Aβ phagocytosis by microglia could produce beneficial outcomes.

A powerful approach to manipulate immune cell function is via genetically encoded chimeric antigen receptors (CARs) that enable antigen-targeted effector responses [8]. While thus far used extensively in systemic immune cells, the potential of this technology has yet to be explored in microglia. Here we generated multiple CAR variants designed to promote microglial phagocytosis and used single-chain variable fragments (scFv) derived from three FDA-approved monoclonal antibodies to target Aβ. We show that expression of anti-amyloid CARs in human iPSC-derived and primary mouse microglia drive a selective increase in engulfment of Aβ42.

## Results

**Fig. 1:**
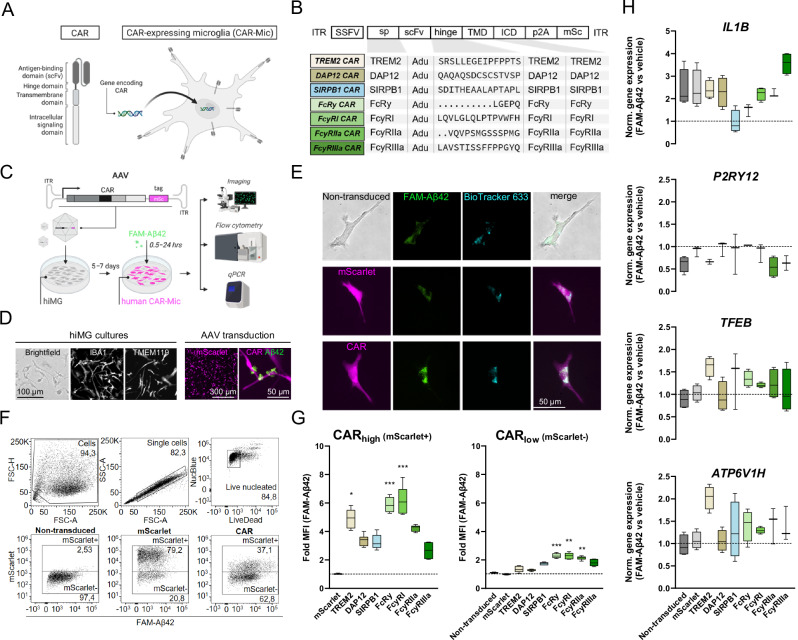
Screening of anti-amyloid CARs in human iPSC-derived microglia (hiMG). **A** schematic showing CAR structure and approach. **B** composition of screened constructs. **C** Outline of the screening procedure. **D** Morphology and marker expression in hiMG. Magenta: mScarlet expression post viral transduction (left) and CAR-Mic targeting of extracellular FAM-Aβ42 (right). **E** Fluorescence images showing intracellular co-localization of Aβ and lysosomes after incubation. **F** flow cytometric gating strategy. **G** Summary of flow cytometric data showing fold MFI values for the mScarlet+ (CAR high) population versus the mScarlet- (CAR low) population (*n *= 4‒6 per group) relative to viral control group (mScarlet). **H** comparative gene expression analysis of activation/lysosomal markers (as indicated) after incubation with FAM-Aβ42 versus vehicle control for 24 h (*n *= 3‒4 per group). *P < 0.05 **P < 0.01, ***P < 0.001.

To construct anti-amyloid CARs, endogenous receptors associated with IgG-dependent or independent Aβ phagocytosis in microglia were used as backbones and combined with a scFv for Aβ targeting (Fig. 1A, B). Seven constructs were assembled and packaged in adeno-associated virus (AAV) particles (Fig. 1C) using a recently developed capsid [9] to support quantitative screening in human iPSC-derived microglia (hiMG) (Fig. 1D). Following a 5‒7 day expression period, the ability of mScarlet+ hiMG (viral control and CARs) to engulf pre-aggregated FAM-labeled Aβ42 was assessed relative to non-transduced cells using a multicolor flow cytometry panel (Fig. 1E, F). No apparent toxicity was observed after application of virus particles over this time period (Fig. S2A–C). Transduction efficiencies, determined as percent mScarlet+ cells, were on average 32.5% for CARs and 70.5% for viral control (expression of mScarlet only) (see Table 1 for details). hiMG expressing anti-amyloid CARs displayed up to 6-fold increases in mean fluorescence intensity (MFI) after a 2-hr incubation period (Fig. 1G). A similar increase was observed in a separate assay of phagocytosis using coated beads (Fig. S3A–C). Gene expression analysis after a 24-hr incubation period with FAM-Aβ42 versus vehicle control showed trends toward upregulation of genes associated with pro-inflammatory activation (*IL1B*), lysosomal induction (*TFEB*) and acidification (*ATP6V1H*) for most of the CARs (Fig. 1H).

**Table 1:** Transduction efficiency data obtained in flow cytometry analyses of hiMG and primary mouse microglia.

Figure 1	Range (%)	Mean	SD	n
Non-transduced	0	0.00	0.00	4
mScarlet	76,9–79,2	78.23	0.90	4
TREM2 CAR	16–20,5	17.95	1.56	6
DAP12 CAR	37,8–39,6	38.62	0.71	6
SIRPB1 CAR	16,4–17,9	17.17	0.49	6
FcRγ CAR	27,2–29,2	27.83	0.68	6
FcγRI CAR	37,1–40,7	38.90	1.33	6
FcγRIIa CAR	46,2–48,8	47.08	0.84	6
FcγRIIIa CAR	45–47,4	46.17	0.99	6

Fig. 2B	Range (%)	Mean	SD	n
Non-transduced	0,18–0,28	0.22	0.03	6
mScarlet	46–53	48.10	2.31	6
Adu FcRγ CAR	15,6–16,4	16.12	0.25	6
Don FcRγ CAR	46,3–50,1	48.00	1.22	6
Lec FcRγ CAR	18,9–24,9	23.36	2.06	6
Pras FcRγ CAR	45,6–48,8	47.82	1.13	6

Fig. 2D	Range (%)	Mean	SD	n
Non-transduced	2,19–2,64	2.47	0.17	4
mScarlet	77,7–79,5	78.50	0.68	4
FcRγ	10,7–13,6	12.52	0.94	6
FcγRIIb ICD	19,6–23	20.80	1.16	6

Fig. 2E	Range (%)	Mean	SD	n
mScarlet	60,7–67,1	64.23	2.65	3
mScarlet CytoD	66,4–69,3	68.03	1.21	3
FcRγ	19,7–23,8	21.10	1.91	3
FcRγ CytoD	18,6–20,5	19.70	0.80	3

Fig. 3	Range (%)	Mean	SD	n
Non-transduced	0	0.00	0.00	4
mScarlet	80,1–91,8	85.88	4.72	4
mFcRγ CAR	50,3–64	56.87	4.93	6
mFcγRI CAR	47,3–59,9	52.62	4.18	6

**Fig. 2:**
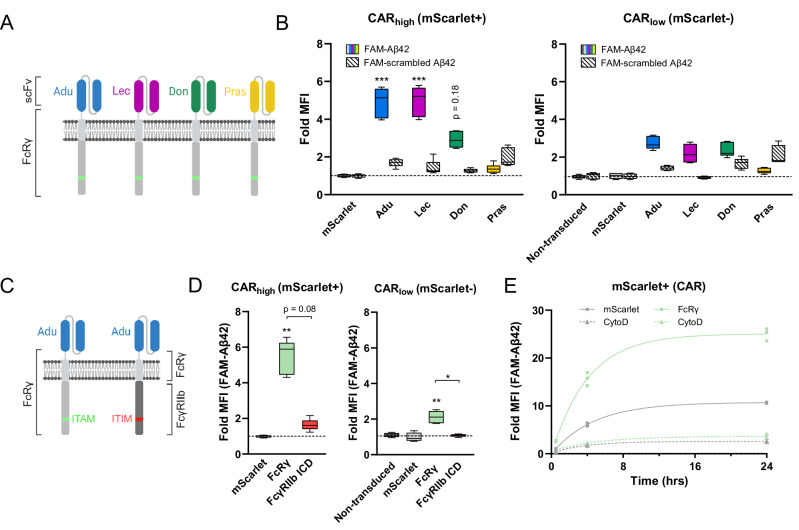
CAR-mediated phagocytosis is dependent on intra- and extracellular domains for selectivity and function. **A** schematic of CAR constructs used for testing of Aβ selectivity. **B** summary of flow cytometric data showing fold MFI values for the mScarlet+ (CAR high) population versus the mScarlet- (CAR low) population (*n *= 6 per group) relative to viral control group (mScarlet). **C** schematic of CAR constructs used for testing ICD dependence. **D** Summary of flow cytometric data showing fold MFI values for the mScarlet+ (CAR high) population versus the mScarlet- (CAR low) population (*n *= 4‒6 per group) relative to viral control group (mScarlet). **E** Kinetics of FAM-Aβ42 phagocytosis and effect of the actin polymerization inhibitor cytochalasin D (CytoD, 5 µM). *P < 0.05 **P < 0.01, ***P < 0.001. Adu; scFv derived from aducanumab, Lec; scFv derived from lecanemab, Don; scFv derived from donanemab, Pras; scFv derived from prasinezumab. ITAM/ITIM; immunoreceptor tyrosine-based activation/inhibitory motif.

To confirm selectivity toward Aβ we used the FcRγ CAR (Fig. 1B) as base and replaced the scFv derived from aducanumab (the first anti-amyloid antibody to receive FDA approval for treatment of AD) with that of two other amyloid-targeting antibodies (lecanemab and donanemab), as well as an α-synuclein-targeting scFv (prasinezumab) (Fig. 2A). We found that all three amyloid-targeting scFvs facilitated phagocytosis of Aβ42, whereas the α-synuclein-targeting scFv did not (Fig. 2B). Selectivity was further addressed using a scrambled Aβ42 peptide, for which phagocytic activity was similar to control for all constructs (Fig. 2B). The dependency of phagocytic enhancement on intracellular domain (ICD) signaling was tested by replacing the FcRγ ICD with that of the inhibitory receptor FcγRIIb (Fig. 2C), which significantly reduced phagocytic activity toward Aβ (Fig. 2D). CAR-mediated phagocytic enhancement further required actin mobilization and increased over 24 h (Fig. 2E).

**Fig. 3:**
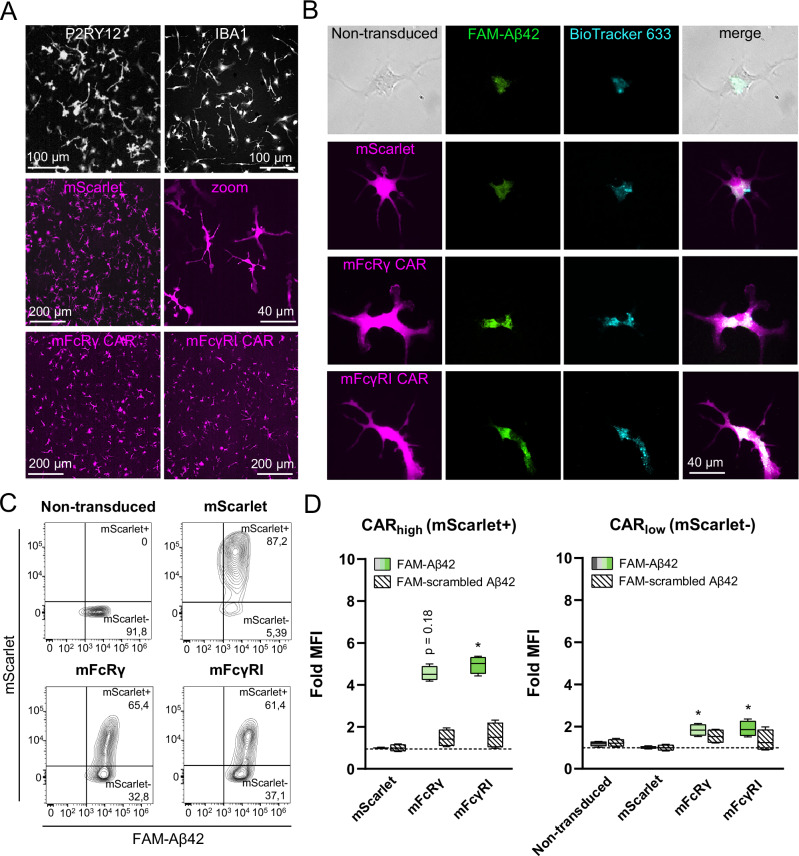
Anti-amyloid CARs promote selective Aβ42 phagocytosis in primary mouse microglia. **A** Immunocytochemistry images showing P2RY12 and IBA1 marker expression (top row). Magenta: mScarlet and CAR expression (as indicated) six days post AAV infection. **B** Fluorescence images showing intracellular co-localization of Aβ and lysosomes after incubation. **C** Flow cytometric analyses. **D** Summary of flow cytometric data showing fold MFI values for the mScarlet+ (CAR high) population versus the mScarlet- (CAR low) population (*n *= 4 per group) relative to viral control group (mScarlet). *P < 0.05.

Finally, we tested murine variants of the two best performing constructs (FcRγ and FcуRI) to confirm CAR function in primary mouse microglia after AAV transduction (Fig. 3A). Following a 2-hr incubation with pre-aggregated FAM-Aβ42 (Fig. 3B) or scrambled FAM-Aβ42, flow cytometric analyses revealed a similar increase in phagocytosis of Aβ42 by mouse CAR-Mic (Fig. 3C, D).

## Discussion

We here report the use of CAR technology in microglia and show that expression of anti-amyloid CARs potently enhances phagocytosis of extracellular Aβ42. Functional screening of multiple constructs in hiMG revealed that TREM2, FcRγ and FcγRI-based CAR variants were most efficient in promoting phagocytic activity. Phagocytic enhancement was largely selective for Aβ42, showed strong dependence on ICD signaling, and was confirmed in primary mouse microglia.

Previous work in macrophages, a systemic immune cell closely related to microglia, has shown that CARs can promote engulfment of diverse targets ranging from tumor cells [10] to Aβ [11], and insights from these studies were leveraged to design CARs for microglia. Given the ontogeny and tissue specialization of microglia, CAR-Mic would likely have a superior ability to survive and operate long-term in the brain parenchyma. However, robust evaluation of this approach requires efficient strategies to generate CAR-Mic in vivo.

While efficient and specific gene delivery to microglia in vivo remains a significant challenge, recent progress in AAV-based approaches [9, 12–14] is providing new opportunities to evaluate genetically engineered microglia in mouse models. A concerted effort to benchmark the growing number of new viral tools would help to further accelerate progress. In addition to gene therapy, human or mouse CAR-Mic could be generated in vitro for subsequent transplantation and replacement of resident cells in the mouse brain [15, 16].

Our findings suggest that equipping microglia in vivo with anti-amyloid CARs could enable efficient Aβ clearance in AD. As compared to current passive immunotherapeutic strategies in clinical use, the approach could support long-term management of amyloid pathology and possibly circumvent side effects associated with systemic antibody delivery (i.e. brain bleeding and swelling) [6, 7, 17]. Moreover, expanding CAR functionality (e.g., through co-induction of lysosomal or neurotrophic signaling pathways) and implementing temporal control of CAR expression could further increase the utility of this approach. Beyond AD, CARs could be modified to target phagocytosis of other misfolded/aggregated proteins, or to promote other microglial functions.

Advanced microglia-based approaches could provide more effective means to modify pathology in NDDs, yet several challenges exist regarding the translation of such approaches to the clinic. Two general strategies may be pursued: (1) targeting of the resident microglial cell population (using a viral or non-viral gene delivery vehicle) or (2) complete or partial cell replacement using transplantation of in vitro-engineered exogenous cells such as stem-cell derived microglia [18, 19]. Regardless of approach, acute and long-term safety, large-scale manufacturing and treatment cost are examples of hurdles that need to be overcome. Despite these challenges, it is imperative that new treatments are developed to address the projected increase in disease burden of NDDs in coming decades. Further research on microglia-based therapies and continued advancements in human disease modeling will be critical for successful clinical translation.

## Materials and methods

### Constructs and virus

Human and mouse codon-optimized DNA sequences were generated from back-translation of UniProt-derived and publicly available monoclonal antibody sequences. All constructs were expressed under the control of the SSFV promoter in a single ORF featuring a P2A-mScarlet sequence for detection. All plasmids (SFFV-CAR-P2A-mScarlet and SFFV-mScarlet) were custom-cloned and packaged into AAV by VectorBuilder (Chicago, IL, USA) using rAAV2/cMG (Addgene #184539). Viral titers ranged from 1.3‒4 × 10^12^ vg/ml for all constructs.

### Peptide preparation and incubation

FAM-labeled Aβ1-42 (Aβ42) and scrambled Aβ42 peptides were purchased from AnaSpec (AS-23525-05, AS-60892). Peptides were briefly solubilized in DMSO at 1 mM, bath sonicated for 5 min and immediately diluted 1:100 in sterile low pH/high salt buffer (150 mM NaCl, 10 mM HCl) to promote amyloid plaque formation [20]. Preparations were either frozen at −80 °C or incubated for 24 h at 37 °C before added to cultures. 5 μl of pre-aggregated peptide (corresponding to a final 500 nM concentration of monomer) was gently pipette-mixed and then added to wells (1:20 dilution in medium). Cells were incubated with peptide at 37 °C for 2 h prior to flow cytometric and image analyses. In experiments involving actin polymerization inhibition, 5 µM cytochalasin D (Sigma-Aldrich, C8273) was added to wells 5 min prior to incubation with Aβ42 for 0.5, 4 or 24 h.

### Human induced pluripotent stem cell (hiPSC)-derived microglia

The commercially available human cell line WTSIi015-A (Sigma-Aldrich) was used and hiPSC-derived microglia were differentiated as previously described [21]. Briefly, hiPSCs were thawed and cultured on Matrigel (Corning) with daily media change. Following embryonic body formation (~2 weeks), cells were transferred to a 6-well plate in hematopoietic medium (HM). After about 3–4 weeks with weekly media changes, primitive macrophage precursors were harvested. Precursor cells were counted and seeded at a density of 3 × 10^4^ cells per well in 96-well plates, using microglia medium containing 2 mM GlutaMAX, penicillin (100 U/ml), streptomycin (100 μg/ml), 55 μM β-mercaptoethanol and human IL-34 and GM-CSF (100 and 10 ng/ml, PeproTech) in Advanced DMEM F12 medium (Gibco). A full medium change was performed every other day. The purity of hiPSC-derived microglia was assessed by determining the percentage of IBA1-positive cells after one week in culture (Fig. S1).

### Primary mouse microglia

Primary P2 microglia isolated from whole brain tissue of C57BL/6 mice were obtained from BrainBits (TransnetYX, USA) and handled according to the supplier’s protocol. Cells were counted and seeded at 2–3 × 10^4^ cells per well in 96-well plates using TIC medium [22] containing TGF-β2 (2 ng/ml, PeproTech), insulin (5 μg/ml, Sigma-Aldrich), cholesterol (1.5 μg/ml, Avanti Polar Lipids), N-acetyl cysteine, apo-transferrin and sodium selenite (5 μg/ml, 100 μg/ml and 100 ng/ml, Sigma-Aldrich), murine IL-34 (100 ng/ml, R&D systems), penicillin (100 U/ml), streptomycin (100 μg/ml) in DMEM/F12 with L-Glutamine (Gibco). Half of the medium volume was replaced every 2–3 days.

### Viral transduction

AAV particles encoding CARs or mScarlet only (viral control) were prediluted in sterile PBS (pH 7.4, Gibco) and applied to hiMG and primary mouse microglial cultures at MOI 2.5‒5 × 10^4^ (7.5 × 10^8^ ‒ 1.5 × 10^9^ vg/100 µl/well). Cultures were incubated with virus or diluent only (control) overnight at 37 °C in presence of 200 nM doxorubicin (Sigma-Aldrich, D1515) [9], and a full medium replacement was performed the next day. Medium changes were thereafter performed every 2–3 days. Cells were allowed 5–7 days to express the transgene prior to analysis.

### Flow cytometry

Following Aβ incubation, single-cell suspensions of hiMG or primary mouse microglia were prepared by detaching the adherent cells for 10 min at 37 °C using 0.25% Trypsin-EDTA (Gibco). After dilution in PBS supplemented with 5% FCS (ThermoFisher) the cells were stained with Live/Dead Fixable Far Red (1:1000, Invitrogen) for 15 min, centrifuged at 300 x g for 5 min and resuspended in PBS. One drop of NucBlue™ (Life Technologies) was added per sample 10 min before acquisition. Data were collected on a LSR Fortessa instrument (BD) and analyzed using the FlowJo™ software (BD Life Sciences). Each replicate (n) consisted of 2–4 pooled wells per group. In all graphs the mean fluorescence intensity values were normalized to mScarlet (viral control).

### Gene expression analysis

Following a 24-hr incubation with Aβ42 (500 nM) or vehicle control (150 mM NaCl, 10 mM HCl, 0.05% DMSO), hiMG were lysed in RLT buffer (Qiagen) with 1 M dithiothreitol (DTT) and stored at −80 °C. RNA extraction was performed using the RNeasy Mini Kit (Qiagen) according to the manufacturer’s instructions. cDNA was prepared using the High-Capacity RNA-to-cDNA Kit (ThermoFisher, #4387406) following the manufacturer’s instructions. Gene expression was analyzed with qPCR using the following primers: *ACTB* (Hs01060665_g1), *IL1B* (Hs01555410_m1), *P2RY12* (Hs01881698_s1), *TFEB* (HS01065085_m1) and *ATP6V1H* (Hs00977530_m1) (ThermoFisher). Expression was calculated using the delta-delta-ct method and normalized to vehicle control.

### Immunocytochemistry and imaging

hiMG (1 week in culture) were washed with Tris-buffered saline solution (TBS), fixated with Histofix (Histolab) for 15 min and incubated in permeabilization buffer (0.3% Triton X-100 in TBS) for 15 min. After 1 hr of blocking (5% goat serum in permeabilization buffer), anti-IBA1 (1:500, Cell Signaling #17198 and Wako #019-19741) and anti-TMEM119 (1:500, proteintech #27585-I-AP) primary antibodies were applied overnight at 4 °C. The next day cells were washed and incubated with secondary antibody (1:2000, goat anti-rabbit AF568 or donkey anti-rabbit AF647, Invitrogen) for 1 hr. During wash with TBS, DAPI (1:1000, D1306, ThermoFisher) was added for 5 min. After final washing the cells were coverslipped and mounted with ProLong Gold antifade reagent (Invitrogen #P36930) onto glass slides. Primary microglia were washed with DPBS (pH 7.4, Gibco), fixated with 4% PFA (in DPBS) for 15 min and permeabilized with 0.3% Triton X-100 (in DPBS) for 10 min. Following blocking for 30 min (2% BSA, 0.1% Triton X-100 in DPBS), anti-IBA1 (1:100, MABN92, Merck) and anti-P2RY12 (1:200, BioLegend #848002) primary antibodies were applied overnight at 4 °C under gentle shaking, washed with DPBS and incubated with goat anti-mouse (1:1000, A21121, Invitrogen) and anti-rat (1:1000, A-11006, Invitrogen) secondary antibodies for 2 h at room temperature. After final washing the cells were coverslipped and mounted onto slides with ProLong Gold (Invitrogen). Intracellular Aβ and lysosomes were imaged live in 96-well plates using BioTracker NIR633 (#SCT138, Sigma-Aldrich) immediately following a 2-hr incubation with FAM-Aβ42. BioTracker dye was prepared according to the manufacturer’s instruction and applied to cells (1:1000) for 30 min at 37 °C. All imaging was performed on Ts2, Ti2-E, and A1 microscopes (Nikon, Japan) using 10, 20 and 40x air objectives together with the NIS-elements BR software.

### Statistics

Statistical significance was evaluated using Kruskal‒Wallis test with Dunn’s post hoc test for multiple comparisons. Asterisks denote level of significance (*P < 0.05, **P < 0.01, ***P < 0.001) relative to mScarlet. Data were analyzed using the GraphPad Prism software (version 10) and are presented in box and whisker plots featuring median and max-min values unless otherwise indicated.

## Supplementary information

Supplementary Material

## Data Availability

All data is included in the article or supplementary material. Amino acid sequences for all constructs are listed in the supplementary material.
