# Correction: Expression of anti-amyloid CARs in microglia promotes efficient and selective phagocytosis of Aβ1‒42

**DOI:** 10.1038/s41434-025-00562-5

**Published:** 2025-08-15

**Authors:** Christina N. Heiss, Rebecca Riise, Eric Hanse, Stefanie Fruhwürth, Henrik Zetterberg, Andreas Björefeldt

**Affiliations:** 1 Department of Psychiatry and Neurochemistry, Institute of Neuroscience and Physiology, University of Gothenburg, Gothenburg, Sweden; 2 Sahlgrenska Center for Cancer Research, Institute of Clinical Sciences, University of Gothenburg, Gothenburg, Sweden; 3 Department of Physiology, Institute of Neuroscience and Physiology, University of Gothenburg, Gothenburg, Sweden; 4 Clinical Neurochemistry Laboratory, Sahlgrenska University Hospital, Mölndal, Sweden; 5 Department of Neurodegenerative Disease, UCL Institute of Neurology, London, UK; 6 UK Dementia Research Institute at UCL, London, UK; 7 Hong Kong Center for Neurodegenerative Diseases, Hong Kong, China; 8 Wisconsin Alzheimer’s Disease Research Center, University of Wisconsin School of Medicine and Public Health, University of Wisconsin-Madison, Madison, WI USA

**Keywords:** Neuroscience, Neurological disorders

Correction to: *Gene Therapy* 10.1038/s41434-025-00534-9, published online 10 April 2025

In this article the Competing Interest statement was previously given as “An international patent application has been filed based on this work (PCT/EP2024/058078). R.R., E.H., H.Z. and A.B. are shareholders in MicThera.” but should be updated to “An international patent application has been filed based on this work (PCT/EP2024/ 058078). R.R., E.H., H.Z. and A.B. are shareholders in CERimmune Therapeutics.”.

This correction is initiated solely on account of the company’s name change.

The original article has been updated.

