# Supplementary material for: Expression of anti-amyloid CARs in microglia promotes efficient and selective phagocytosis of Aβ1‒42
Source: Gene Ther. 2025 Apr 10;32(4):333–8. doi: 10.1038/s41434-025-00534-9 (PMC12310543; doi:10.1038/s41434-025-00534-9)
Supplement: Supplementary file 1 — Supplementary Material [file 41434_2025_534_MOESM1_ESM.pdf]

Supplementary Material

Expression of anti-amyloid CARs in microglia promotes efficient and selective phagocytosis of Aβ1–42

Christina Heiss, Rebecca Riise, Eric Hanse, Stefanie Fruhwürth, Henrik Zetterberg and Andreas Björefeldt

Figure S1

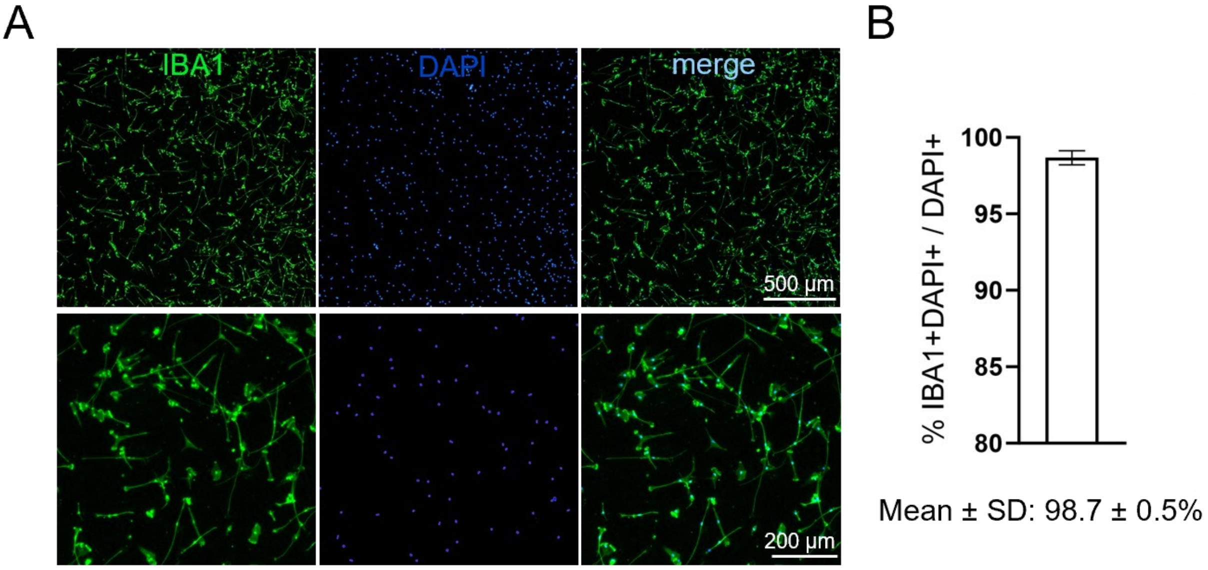

Quantification of IBA1-postitive cells in hiPSC-derived (hiMG) microglial cultures. (A) immunocytochemistry images showing IBA1 and DAPI stains performed after 5 days in culture (magnified images in bottom row). (B) average percentage of double-postitive (IBA1+DAPI+) cells over total cells (DAPI+) after differentiation (n = 3 replicates, one 2.2 x 2.2 mm image quantified per replicate, total of 2125 analyzed cells).

Figure S2

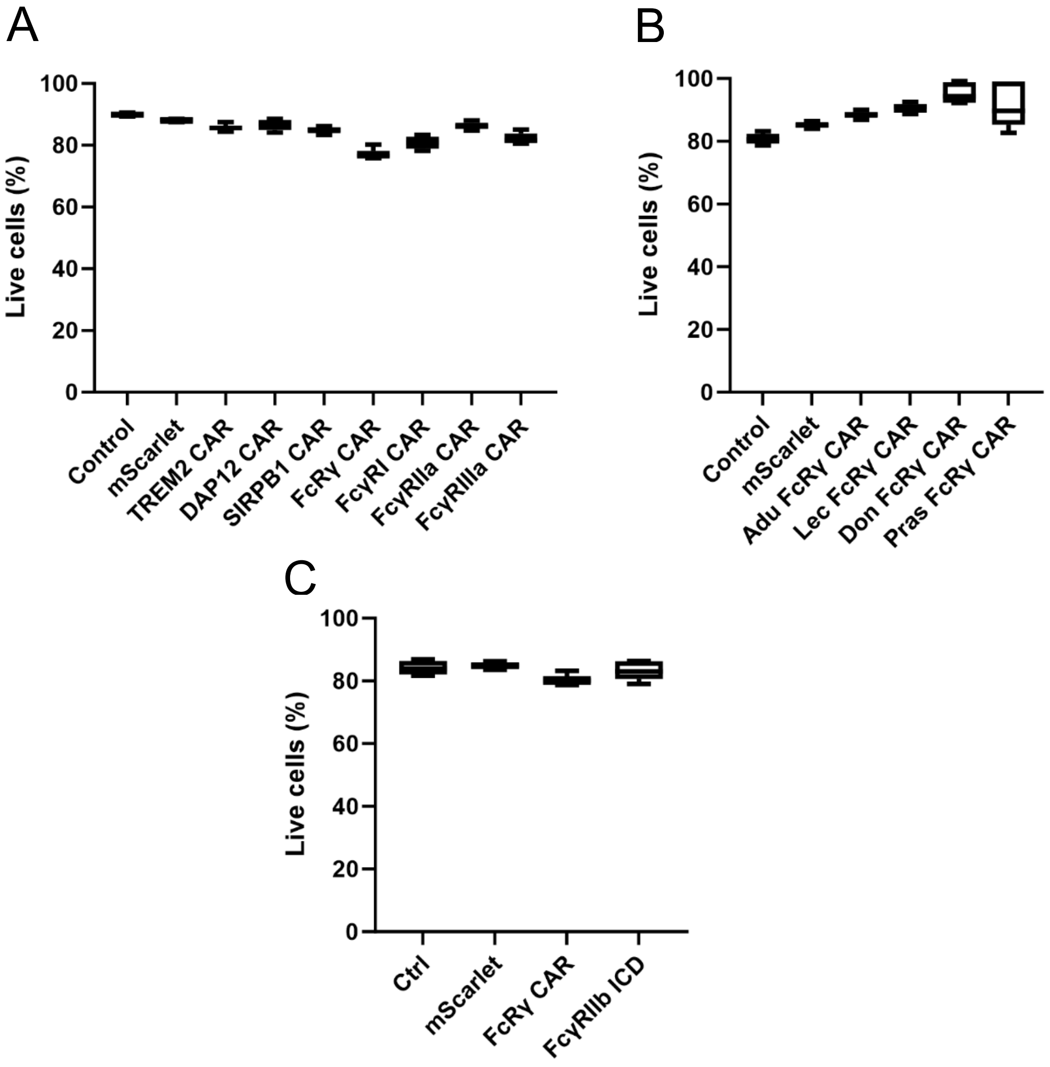

No apparent toxicity after AAV-cMG transduction with mScarlet (viral control) or CARs in hiMG cultures 5–7 days post infection. (A) percent live cells detected using flow cytometry analysis of Live/Dead marker in dataset derived from Fig. 1G. (B) percent live cells detected using flow cytometry analysis of Live/Dead marker in dataset derived from Fig. 2B. (C) percent live cells detected using flow cytometry analysis of Live/Dead marker in dataset derived from Fig. 2D. N = 4–6 replicates per group.

Figure S3

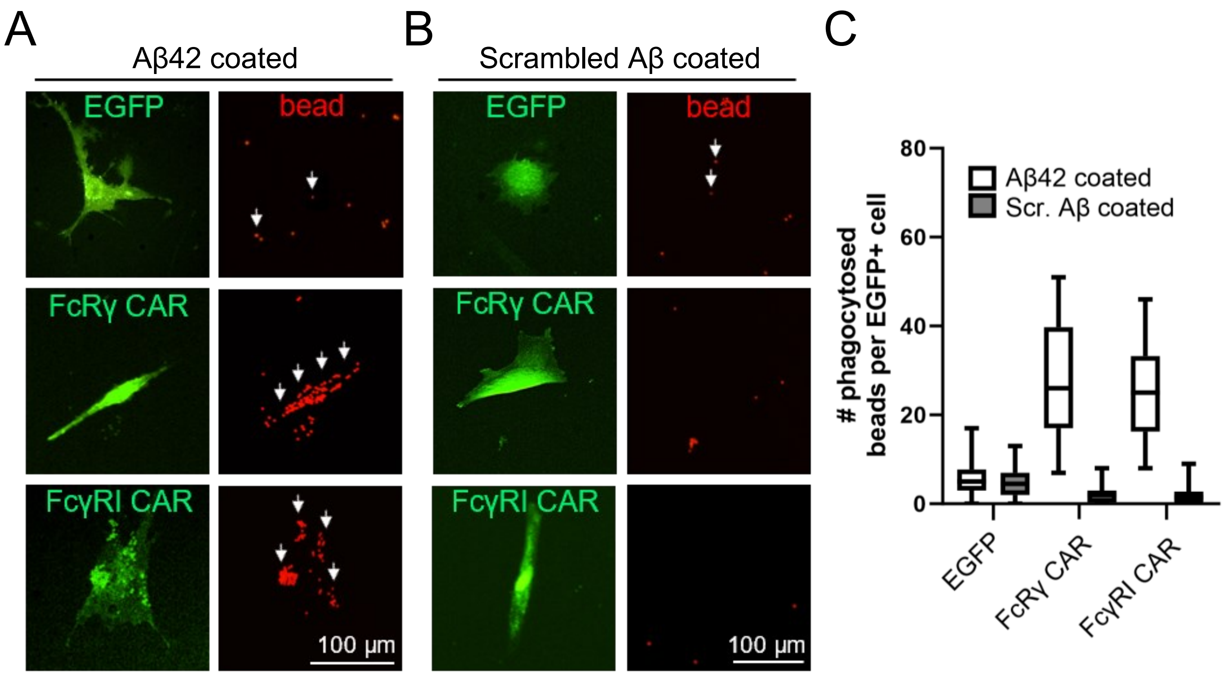

Coated bead engulfment assay in hiMG following AAV2-mediated transduction. (A) representative immunocytochemistry images showing engulfment of Aβ42 coated polystyrene beads (red) by hiMG expressing EGFP or CAR (green) as indicated. (B) representative immunocytochemistry images showing engulfment of scrambled Aβ coated polystyrene beads (red) by hiMG expressing EGFP or CAR (green) as indicated. (C) Summary graph showing number of phagocytosed beads between groups (n = 20 EGFP+ cells per group).

Supplementary methods

Immunocytochemistry, imaging and analysis. hiMG were seeded onto 12 mm round coverslips (#1.5, 630-2190, VWR) in a 24-well plate and cultured for 5 days as described in main methods section. Cells were washed once in DPBS (Gibco) and fixated in 2% PFA in DPBS for 20 minutes, then permeabilized and blocked for 10 minutes (2% BSA, 0.3% Triton X-100 in DPBS) and incubated with an anti-IBA1 primary antibody (1:500, Wako #019-19741) for 1 hr. After wash, cells were incubated with a donkey anti-rabbit AF647 secondary antibody (1:2000, A31573, Invitrogen) for 1 hr at room temperature. After final wash coverslips were mounted onto slides using ProLong Gold with DAPI stain (P36931, Invitrogen) and imaged on a Ti2-E microscope (Nikon, Japan). Microglial purity was quantified in ImageJ as the percentage of IBA1+DAPI+ cells / total DAPI+ cells using the threshold, image calculator and particle analysis functions.

Cell viability analysis. Prior to flow cytometry analysis, cells were stained with Live/Dead Fixable Far Red (1:1000, Invitrogen) for 15 min, centrifuged at 300 x g for 5 minutes and resuspended in PBS. One drop of NucBlue™ (Life Technologies) was added per sample 10 min before acquisition. The percentage of live nucleated cells was quantified and compared between groups.

AAV2 transduction. Viral particles carrying CARs or EGFP only (viral control) were packaged by VectorBuilder (Chicago, IL, USA) using AAV2 rep/cap. Titers ranged from 9×10^{11} – 1.5×10^{12} vg/ml. Virus was prediluted in sterile PBS (pH 7.4) and applied to hiMG at MOI 2.5×10^{4} (7.5×10^{8} vg/100µl/well). hiMG cultures (differentiated and maintained as described in main methods section) were incubated with AAV2 overnight at 37 °C and a full medium replacement was performed the next day. Medium changes were thereafter performed every 2-3 days. Cells were allowed 5 days to express the transgene prior to analysis.

Bead engulfment assay. Carboxyl microspheres (1 µm diameter, 0.9 mg/ml) were purchased from Bangs Laboratories (FCFR006/ FC04F) and prepared for coating according to the manufacturer's instructions. Briefly, COOH groups were activated using MES buffer (Sigma, M3671) containing water-soluble carbodiimide (5 mM, Sigma #03449) and sulfo-NHS (5 mM, Sigma #56485). Beads were washed in coupling buffer (PBS, pH 7.4, Gibco) and mixed with Aβ42 or scrambled Aβ peptide (AnaSpec, AS-20276 and AS-25382) at 2.5x concentration of calculated monolayer (20 µg peptide/mg bead) for 2 hrs at room temperature. Excess peptide was discarded by centrifugation at 5500 x g for 3 minutes. Coated beads were resuspended in quenching buffer containing 30 mM ethanolamine (Sigma, E9508) and 0.1% BSA (in PBS) for 30 minutes, washed and stored in PBS containing 0.05% BSA at 4°C (see Bangs Laboratories TechNote 205 for additional details). Within one week of preparation, 1×10^{6} beads per well were added to hiMG and incubated at 37 °C for 1 hr. Cells were washed in DPBS, immediately fixated in 4% PFA (in DPBS) for 20 minutes, and washed a final time. Beads and cells were imaged on a Ti2-E microscope (Nikon, Japan) using 480 nm (GFP) and 625 nm (Cy5) excitation. The number of engulfed beads per EGFP+ cell was quantified in Image J.

List of amino acid sequences

Signal peptide scFv aducanumab Hinge domain TM domain Intracellular signaling domain

Human TREM2 CAR
MEPLRLLILLFVTELSGAQVQLVESGGGVVQPGRSLRLSCAASGFAFSSYGMHWVRQAPGKGLEWV
AVIWFDGTKKYYTDSVKGRFTISRDNSKNTLYLQMNTLRAEDTAVYYCARDRGIGARRGPYYMDVWG
KGTTVTVSGGGGSGGGGSGGGGSDIQMTQSPSSLSASVGDRVTITCRASQSISSYLNWYQQKPGKA
PKLLIYAASSLQSGVPSRFSGSGSGTDFTLTISSLQPEDFATYYCQQSYSTPLTFGGGTKVEIKSRSLL
EGEIPFPPTSILLLLACIFLIKILAASALWAAAWHGQKPGTHPPSELDCGHDPGYQLQTLPGLRDT

Human DAP12 CAR
MGGLEPCSRLLLLPLLLAVSGQVQLVESGGGVVQPGRSLRLSCAASGFAFSSYGMHWVRQAPGKGL
EWVAVIWFDGTKKYYTDSVKGRFTISRDNSKNTLYLQMNTLRAEDTAVYYCARDRGIGARRGPYYMD
VWGKGTTVTVSGGGGSGGGGSGGGGSDIQMTQSPSSLSASVGDRVTITCRASQSISSYLNWYQQK
PGKAPKLLIYAASSLQSGVPSRFSGSGSGTDFTLTISSLQPEDFATYYCQQSYSTPLTFGGGTKVEIKQ
AQAQSDCSCSTVSPGVLAGIVMGDLVLTVLIALAVYFLGRLVPRGRGAAEAATRKQRITETESPYQEL
QGQRSDVYSDLNTQRPYYK

Human SIRPB1 CAR
MPVPASWPHLPSPFLLMTLLLGRLTGVAGQVQLVESGGGVVQPGRSLRLSCAASGFAFSSYGMHWV
RQAPGKGLEWVAVIWFDGTKKYYTDSVKGRFTISRDNSKNTLYLQMNTLRAEDTAVYYCARDRGIGA
RRGPYYMDVWGKGTTVTVSGGGGSGGGGSGGGGSDIQMTQSPSSLSASVGDRVTITCRASQSISS
YLNWYQQKPGKAPKLLIYAASSLQSGVPSRFSGSGSGTDFTLTISSLQPEDFATYYCQQSYSTPLTFG
GGTKVEIKSDITHEAALAPTAPLLVALLLGPKLLLVVGVSAIYICWKQKA

Human FcRγ CAR
MIPAVVLLLLLLVEQAAAQVQLVESGGGVVQPGRSLRLSCAASGFAFSSYGMHWVRQAPGKGLEWV
AVIWFDGTKKYYTDSVKGRFTISRDNSKNTLYLQMNTLRAEDTAVYYCARDRGIGARRGPYYMDVWG
KGTTVTVSGGGGSGGGGSGGGGSDIQMTQSPSSLSASVGDRVTITCRASQSISSYLNWYQQKPGKA
PKLLIYAASSLQSGVPSRFSGSGSGTDFTLTISSLQPEDFATYYCQQSYSTPLTFGGGTKVEIKLGEPQ
LCYILDAILFLYGIVLTLLYCRLKIQVRKAAITSYEKSDGVYTGLSTRNQETYETLKHEKPPQ

Human FcγRI CAR
MWFLTTLLLWVPVDGQVQLVESGGGVVQPGRSLRLSCAASGFAFSSYGMHWVRQAPGKGLEWVA
VIWFDGTKKYYTDSVKGRFTISRDNSKNTLYLQMNTLRAEDTAVYYCARDRGIGARRGPYYMDVWGK
GTTVTVSGGGGSGGGGSGGGGSDIQMTQSPSSLSASVGDRVTITCRASQSISSYLNWYQQKPGKAP
KLLIYAASSLQSGVPSRFSGSGSGTDFTLTISSLQPEDFATYYCQQSYSTPLTFGGGTKVEIKLQVLGL
QLPTPVWFHVLFYLAVGIMFLVNTVLWVTIRKELKRKKKWDLEISLDSGHEKKVISSLQEDRHLEEELK
CQEQKEEQLQEGVHRKEPQGAT

Human FcγRIIa CAR
MTMETQMSQNVCPRNLWLLQPLTVLLLLASADSQVQLVESGGGVVQPGRSLRLSCAASGFAFSSYG
MHWVRQAPGKGLEWVAVIWFDGTKKYYTDSVKGRFTISRDNSKNTLYLQMNTLRAEDTAVYYCARD
RGIGARRGPYYMDVWGKGTTVTVSGGGGSGGGGSGGGGSDIQMTQSPSSLSASVGDRVTITCRAS
QSISSYLNWYQQKPGKAPKLLIYAASSLQSGVPSRFSGSGSGTDFTLTISSLQPEDFATYYCQQSYST
PLTFGGGTKVEIKVQVPSMGSSSPMGIIVAVVIATAVAAIVAAVVALIYCRKKRISANSTDPVKAAQFEP
PGRQMIAIRKRQLEETNNDYETADGGYMTLNPRAPTDDDKNIYLTLPPNDHVNSNN

Human FcγRIIIa CAR
MWQLLLPTALLLLVSAQVQLVESGGGVVQPGRSLRLSCAASGFAFSSYGMHWVRQAPGKGLEWVA
VIWFDGTKKYYTDSVKGRFTISRDNSKNTLYLQMNTLRAEDTAVYYCARDRGIGARRGPYYMDVWGK
GTTVTVSGGGGSGGGGSGGGGSDIQMTQSPSSLSASVGDRVTITCRASQSISSYLNWYQQKPGKAP
KLLIYAASSLQSGVPSRFSGSGSGTDFTLTISSLQPEDFATYYCQQSYSTPLTFGGGTKVEIKITQGLAV
STISSFFPPGYQVSFCLVMVLLFAVDTGLYFSVKTNIRSSTRDWKDHKFKWRKDPQDK

Human FcRγ/FcγRIIb CAR (ICD from FcγRIIb)
MIPAVVLLLLLLVEQAAAQVQLVESGGGVVQPGRSLRLSCAASGFAFSSYGMHWVRQAPGKGLEWV
AVIWFDGTKKYYTDSVKGRFTISRDNSKNTLYLQMNTLRAEDTAVYYCARDRGIGARRGPYYMDVWG
KGTTVTVSGGGGSGGGGSGGGGSDIQMTQSPSSLSASVGDRVTITCRASQSISSYLNWYQQKPGKA
PKLLIYAASSLQSGVPSRFSGSGSGTDFTLTISSLQPEDFATYYCQQSYSTPLTFGGGTKVEIKLGEPQ
LCYILDAILFLYGIVLTLLYCVVALIYCRKKRISALPGYPECREMGETLPEKPANPTNPDEADKVGAENTI
TYSLLMHPDALEEPDDQNRI

Mouse FcRγ CAR
MISAVILFLLLLVEQAAAQVQLVESGGGVVQPGRSLRLSCAASGFAFSSYGMHWVRQAPGKGLEWVA
VIWFDGTKKYYTDSVKGRFTISRDNSKNTLYLQMNTLRAEDTAVYYCARDRGIGARRGPYYMDVWGK
GTTVTVSGGGGSGGGGSGGGGSDIQMTQSPSSLSASVGDRVTITCRASQSISSYLNWYQQKPGKAP
KLLIYAASSLQSGVPSRFSGSGSGTDFTLTISSLQPEDFATYYCQQSYSTPLTFGGGTKVEIKLGEPQL
CYILDAVLFLYGIVLTLLYCRLKIQVRKAAIASREKADAVYTGLNTRSQETYETLKHEKPPQ

Mouse FcγRI CAR
MILTSFGDDMWLLTTLLLWVPVGGQVQLVESGGGVVQPGRSLRLSCAASGFAFSSYGMHWVRQAP
GKGLEWVAVIWFDGTKKYYTDSVKGRFTISRDNSKNTLYLQMNTLRAEDTAVYYCARDRGIGARRGP
YYMDVWGKGTTVTVSGGGGSGGGGSGGGGSDIQMTQSPSSLSASVGDRVTITCRASQSISSYLNW
YQQKPGKAPKLLIYAASSLQSGVPSRFSGSGSGTDFTLTISSLQPEDFATYYCQQSYSTPLTFGGGTK
VEIKLQVLGPQSSAPVWFHILFYLSVGIMFSLNTVLYVKIHRLQREKKYNLEVPLVSEQGKKANSFQQV
RSDGVYEEVTATASQTTPKEAPDGPRSSVGDCGPEQPEPLPPSDSTGAQTSQS

Lecanemab scFv
EVQLVESGGGLVQPGGSLRLSCSASGFTFSSFGMHWVRQAPGKGLEWVAYISSGSSTIYYGDTVKG
RFTISRDNAKNSLFLQMSSLRAEDTAVYYCAREGGYYYGRSYYTMDYWGQGTTVTVSSGGGGSGG
GGSGGGGSDVVMTQSPLSLPVTPGAPASISCRSSQSIVHSNGNTYLEWYLQKPGQSPKLLIYKVSNR
FSGVPDRFSGSGSGTDFTLRISRVEAEDVGIYYCFQGSHVPPTFGPGTKLEIK

Donanemab scFv
QVQLVQSGAEVKKPGSSVKVSCKASGYDFTRYYINWVRQAPGQGLEWMGWINPGSGNTKYNEKFK
GRVTITADESTSTAYMELSSLRSEDTAVYYCAREGITVYWGQGTTVTVSSGGGGSGGGGSGGGGSD
IVMTQTPLSLSVTPGQPASISCKSSQSLLYSRGKTYLNWLLQKPGQSPQLLIYAVSKLDSGVPDRFSG
SGSGTDFTLKISRVEAEDVGVYYCVQGTHYPFTFGQGTKLEIK

Prasinezumab scFv
EVQLVESGGGLVQPGGSLRLSCAASGFTFSNYGMSWVRQAPGKGLEWVASISSGGGSTYYPDNVK
GRFTISRDDAKNSLYLQMNSLRAEDTAVYYCARGGAGIDYWGQGTLVTVSSGGGGSGGGGSGGGG
SDIQMTQSPSSLSASVGDRVTITCKSIQTLLYSSNQKNYLAWFQQKPGKAPKLLIYWASIRKSGVPSRF
SGSGSGTDFTLTISSLQPEDLATYYCQQYYSYPLTFGGGTKLEIK
